# Effects of drought and meteorological forcing on carbon and water fluxes in Nordic forests during the dry summer of 2018

**DOI:** 10.1098/rstb.2019.0516

**Published:** 2020-09-07

**Authors:** Anders Lindroth, Jutta Holst, Maj-Lena Linderson, Mika Aurela, Tobias Biermann, Michal Heliasz, Jinshu Chi, Andreas Ibrom, Pasi Kolari, Leif Klemedtsson, Alisa Krasnova, Tuomas Laurila, Irene Lehner, Annalea Lohila, Ivan Mammarella, Meelis Mölder, Mikaell Ottosson Löfvenius, Matthias Peichl, Kim Pilegaard, Kaido Soosar, Timo Vesala, Patrik Vestin, Per Weslien, Mats Nilsson

**Affiliations:** 1 Department of Physical Geography and Ecosystem Science, Lund University, Lund, Sweden; 2 Finnish Meteorological Institute, Helsinki, Finland; 3 Centre for Environmental and Climate Research, Lund University, Lund, Sweden; 4 Department of Forest Ecology and Management, The Swedish University of Agricultural Sciences, Umeå, Sweden; 5 Department of Environmental Engineering, Technical University of Denmark, Lyngby, Denmark; 6 Institute for Atmospheric and Earth System Research, Helsinki University, Helsinki, Finland; 7 Department of Earth Sciences, University of Gothenburg, Gothenburg, Sweden; 8 Institute of Ecology and Earth Sciences, University of Tartu, Tartu, Estonia

**Keywords:** evaporation, surface conductance, net ecosystem productivity, ecosystem respiration, gross primary productivity, heterotrophic respiration

## Abstract

The Nordic region was subjected to severe drought in 2018 with a particularly long-lasting and large soil water deficit in Denmark, Southern Sweden and Estonia. Here, we analyse the impact of the drought on carbon and water fluxes in 11 forest ecosystems of different composition: spruce, pine, mixed and deciduous. We assess the impact of drought on fluxes by estimating the difference (anomaly) between year 2018 and a reference year without drought. Unexpectedly, the evaporation was only slightly reduced during 2018 compared to the reference year at two sites while it increased or was nearly unchanged at all other sites. This occurred under a 40 to 60% reduction in mean surface conductance and the concurrent increase in evaporative demand due to the warm and dry weather. The anomaly in the net ecosystem productivity (NEP) was 93% explained by a multilinear regression with the anomaly in heterotrophic respiration and the relative precipitation deficit as independent variables. Most of the variation (77%) was explained by the heterotrophic component. Six out of 11 forests reduced their annual NEP with more than 50 g C m^−2^ yr^−1^ during 2018 as compared to the reference year. The NEP anomaly ranged between −389 and +74 g C m^−2^ yr^−1^ with a median value of −59 g C m^−2^ yr^−1^.

This article is part of the theme issue ‘Impacts of the 2018 severe drought and heatwave in Europe: from site to continental scale’.

## 1. Introduction

Forests play an important role in the global carbon cycle by absorbing about 29% of anthropogenic emissions [1]. Maintaining this sink capacity is accordingly very important in order to limit the increase of carbon dioxide in the atmosphere. The forest sink is however vulnerable to disturbances such as storms, fires, insect infestation, diseases and management, but also to extreme weather events such as excessive heat, drought or flooding [2–5]. Extreme weather events are projected to increase in frequency in the future because of higher temperatures and intensified hydrological cycles [6]. Thus, it is important to understand how disturbances and extreme weather are affecting the carbon exchanges in forests.

Drought is typically a consequence of longer periods of warm and sunny weather leading to high evaporative demand on ecosystems. The effects of drought are multifaceted: forest trees change their use and allocation of nutrients which in turn affects exchanges of carbon dioxide and evaporation [4], both ecosystem respiration (Reco) and gross primary productivity (GPP) are affected with different responses in deciduous and coniferous stands, as demonstrated in North American studies [7–9]. The net effect on the carbon balance also depends on the timing and severity of drought events [10]. Warm and sunny weather during spring, when water supply is adequate, can also compensate for reduced carbon uptake later in the season leaving the annual net ecosystem productivity (NEP; positive when ecosystem gains carbon) unchanged [11,12].

An example of the effect of a severe drought on carbon fluxes is shown by the European heatwave that occurred in 2003. Ciais *et al*. [13] used carbon dioxide flux measurements, crop yields, remote sensing and modelling to assess continental-scale changes in GPP and NEP and found a 30% reduction in GPP which, combined with a smaller decrease in Reco, resulted in a net source of carbon dioxide to the atmosphere. The impact was highly significant, reversing the effect of the four preceding years of net carbon uptake. It can be noted that the decline in respiration occurred in spite of the increase in temperature, being in accordance with autotrophic respiration being mainly controlled by photosynthate production [14]. Reichstein *et al*. [15] also analysed water use efficiency (WUE, defined as GPP/evaporation (E)) for local flux sites in 2003 and found a small decrease in WUE for most sites indicating a larger effect on GPP than on E by the drought.

Von Buttlar *et al*. [16] analysed the impacts of drought and high temperature and its timing on carbon exchange across a range of ecosystems and climatic zones using flux data from 102 sites. They did not find large differences in responses of the plant functional types: evergreen broadleaf, evergreen needle, deciduous and mixed forests. Dryness without extraordinary heat reduced both GPP and Reco which resulted in a small impact on NEP. Drought in combination with high temperature reduced GPP more than Reco, especially noticeable for evergreen broadleaf forests, but the timing of these events was crucial for how they impacted the fluxes. However, the spread in the data was large, and it was therefore difficult to obtain conclusive evidence of differences among forest types. Noormets *et al*. [17] found that even a moderate drought during the period between bud break and full leaf expansion reduced annual NEP by 40% in a mixed oak and maple forest in the USA. The reduction in NEP was caused by 16% reduction in GPP and 11% in Reco. In the boreal region, Grant *et al*. [18] used flux data from nine forests spread across Canada and they concluded that a 3-year long-lasting drought adversely affected well-drained broadleaf forests but not poorly drained conifer forests. They attributed this difference to the lower evaporation rate in the conifers and to subsurface water recharge in the poorly drained soils.

A regional analysis of Canada's boreal forests based on biomass increment by Ma *et al*. [19] found a decreasing sink in the Western region but no such effect in the Eastern region, over the time span 1963 to 2008. They concluded that the decreasing sink was caused by changes in the climate of which drought-induced water stress was the dominating cause. They expressed concern that large parts of Canada's forests could turn into carbon sources if drought conditions continued to intensify in the future. Similar conclusions were reached by Walker *et al*. [20] who found widespread drought-induced stress on black spruce in the interior of Alaska. They used stable isotopes from tree rings for their analyses.

The aim of this study is to better understand the impact of drought on the annual forest carbon and water fluxes in boreal, hemi-boreal and temperate forests in Scandinavia and in the Baltic region. This region experienced severe drought in 2018 which, in several locations, was even stronger than the drought that occurred in central Europe in 2003. We used eddy covariance flux measurements from 11 forests in the region of which three are spruce, three are pine, four are mixed and one is a beech forest. We base the analyses on comparison between selected reference years (without drought) and 2018. We analyse the anomalies in the main carbon fluxes in relation to expected change drivers such as surface conductance, evaporative demand, precipitation deficit and heterotrophic respiration.

## 2. Material and methods

### (a) Sites and flux data

**Figure 1.**
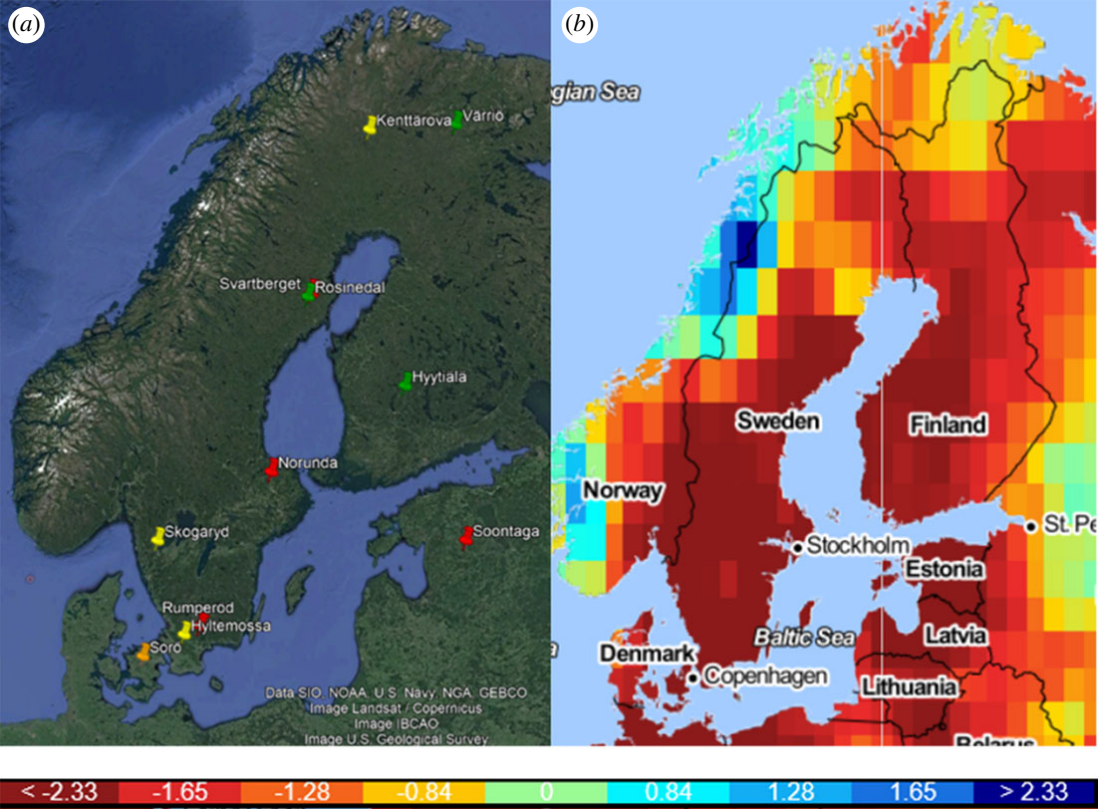
(*a*) Geographic locations of flux sites; spruce—yellow, pine—green, mixed forests—red and beech—orange. (*b*) Map of SPEI 6-month drought index from Global Drought Monitor (https://spei.csic.es/map/maps.html) [21] during the summer of 2018 (April–September). Bottom: SPEI drought scale where dark red is the most severe drought conditions. (Online version in colour.)

The location of the study sites and the drought index, SPEI—Standardized Precipitation Evapotranspiration Index (https://spei.csic.es/index.html), are shown on maps of the Nordic region (figure 1). Basic information about the sites and flux instrumentation are presented in electronic supplementary material, table S3 and S4, respectively. Calculation of half-hourly flux data as well as quality control and assurance were made by the site principal investigators (PIs). In addition to the data from 2018, each site provided 3–4 years of data without obvious effects of drought or other disturbances according to judgement by PIs. Gap filling and partitioning of net fluxes in ecosystem respiration and gross primary production were made using the Jena tool [22] for all sites except for Hyytiälä and Värriö for which in-house software was used. Data filtering based on friction velocity (u_*_) was applied to all sites in order to exclude periods of low turbulence conditions and potential underestimation of measured fluxes. We used both the standardized precipitation evapotranspiration index [21] and precipitation deficit (see below) to characterize the drought conditions.

### (b) Data analyses

#### (i) Growing season data

The definition of growing season was generalized to 1 April to 30 September for the southernmost sites Sorø, Hyltemossa, Rumperöd and Skogaryd. For all other sites, it was defined as 1 May to 31 August.

Measured half-hourly flux data under well-mixed conditions (friction velocity, u_*_ greater than 0.3 m s^−1^) were used for the calculation of variables that were expected to have an impact on responses to drought. The surface conductance, *Gs* (m s^−1^), was calculated for dry daytime conditions using the Penman–Monteith equation [23] omitting energy storage by:

2.1 $$G_{s}=\;\frac{R_{n}\cdot \gamma }{\rho \cdot c_{p}\cdot \delta e(1+\beta )+R_{n}(\Delta \cdot \beta -\gamma )\cdot r_{a}},$$

where *R_n_* is the net radiation (W m^−2^), *γ* is the psychrometric constant (Pa K^−1^), *ρ* is the air density (g m^−3^), *c_p_* is the specific heat at constant pressure (J g^−1^ K^−1^), *δe* is the water vapour pressure deficit (Pa), Δ is the slope of the saturation vapour pressure curve at air temperature (Pa K^−1^), *β* is the Bowen ratio (−) and *r_a_* is the aerodynamic resistance under near-neutral conditions (s m^−1^). The *r_a_* was calculated as [24]

2.2*a* $$r_{a}=\;\frac{u_{z}}{u_{*}^{2}}+r_{b}$$

and

2.2*b* $$r_{b}=6.2\cdot u_{*}^{-2/3},$$

where *u_z_* is wind speed (m s^−1^) at a reference height in the atmospheric boundary layer, *r_b_* is excess resistance (s m^−1^). Here, we omitted the stability correction of *r_a_* because the aerodynamic resistance is typically an order of magnitude smaller than the surface resistance.

The evaporative demand, ED (W m^−2^), or potential evaporation, which gives an integrated measure of the meteorological forcing by radiation, temperature, air humidity and wind speed was calculated from the Monteith equation [23] with zero surface resistance:

2.3 $$ED=\frac{R_{n}\cdot \Delta +\rho \cdot c_{p}\cdot \delta e/r_{a}}{\Delta +\gamma }.$$

We took the growing season relative precipitation deficit (RPD) between 30-year averages and 2018 from the nearest weather station (electronic supplementary material, table S1) to be the most robust drought index that was expected to have an impact on carbon fluxes. This was made for all sites except Skogaryd where the 2018 precipitation from nearby weather stations was found not to be representative of the local conditions. Because of problems with precipitation measurements, we used the relative change in water runoff (L. Klemedtsson 2019, personal communication) between reference year and 2018 as a proxy for precipitation deficit for this site. For all other sites, RPD was calculated as

2.4 $$RPD=\frac{\left(P_{c}-P_{2018}\right)}{P_{c}},$$

where *P_c_* is the 30-year (1981–2010) average sum during growing season and *P*_2018_ is the corresponding sum for 2018.

#### (ii) Annual data

We defined NEP and GPP as positive when the ecosystem is gaining carbon, and accordingly, ecosystem respiration (Reco) as positive when the ecosystem is losing carbon. Thus

2.5 NEP=GPP−Reco.

We then estimated autotrophic respiration (*Ra*) following Litton *et al*. [25] as

2.6 Ra=0.57⋅GPP,

and heterotrophic respiration (*Rh*) as:

2.7 Rh=Reco−Ra.

The impact of the dry and warm season on carbon fluxes and evaporation was evaluated by estimating anomalies between the selected reference year and 2018. The anomaly in flux X was calculated as

2.8 X diff=X (2018)−X (reference year).

If more than one year was accepted as a reference, we used the mean of those years and we also denote such an average—‘reference year'. The reference years are listed in electronic supplementary material, table S2. The selection of reference year was based on the cumulated difference in precipitation between the 30-year average for the nearest weather station and the actual year for the period from beginning of the year until the end of the growing season. The initial aim was to find reference years without precipitation deficit during this period but it turned out to be difficult. For all sites, we found reference years with precipitation deficit less than 13 mm for the period specified above excepting Kenttärova (29 mm), Hyytiälä (30 mm) and Sorø (41 mm). At all of these sites, however, the deficit occurred at the very end of their respective growing seasons. In addition, all three sites also had another year with negligible deficit and we therefore accepted them as reference years.

## 3. Results

### (a) Weather and water relations

The mean monthly air temperature during the growing season of 2018 was higher than the 30-year average at all sites (electronic supplementary material, figure S1). The southernmost site had winter temperatures slightly above or close to zero and maximum monthly summer temperatures reaching 20°C while the corresponding values for the northernmost sites were between −10 and −15°C and 15–20°C, respectively. The site-specific seasonal course of air temperature, soil moisture and respiration components for reference year and 2018 are shown in electronic supplementary material, figure S2a–f.

The monthly SPEI varied between sites (electronic supplementary material. figure S1) and it should be pointed out that this is an average index for the grid where each site is located. The spatial resolution of the grids is 1°, and therefore, it does not give a precise value at the exact location of each site. The precipitation-based drought index, RPD, varied between 0.55 for Skogaryd and 0.10 for Kenttärova (table 1).

**Table 1.** Relative precipitation deficit during growing season for all sites. The site name acronym is also shown in the table.

site (acronym)	RPD (−)
Hyltemossa (Htm)	0.52
Skogaryd (Skg)	0.55
Kenttärova (Ken)	0.1
Hyytiälä (Hyy)	0.32
Rosinedal (Ros)	0.38
Värriö (Var)	0.22
Rumperöd (Rum)	0.47
Soontaga (Son)	0.45
Norunda (Nor)	0.36
Svartberget (Svb)	0.38
Sorø (Sor)	0.38

**Figure 2.**
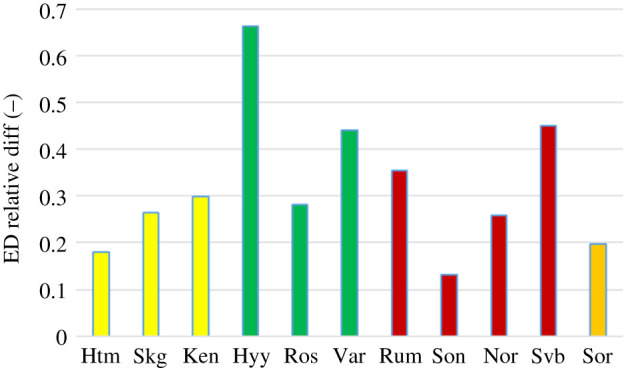
The relative difference in mean growing season evaporative demand (ED) between year 2018 and the reference year. Colour code: yellow—spruce, green—pine, red—mixed and orange—beech. (Online version in colour.)

The mean growing season evaporative demand was higher during the drought year as compared to the reference year for all sites but with quite large variation (figure 2). Hyltemossa, Soontaga and Sorø showed a relatively modest increase with about 12–20% while it was very high for Hyytiälä with an excess demand of 66%.

**Figure 3.**
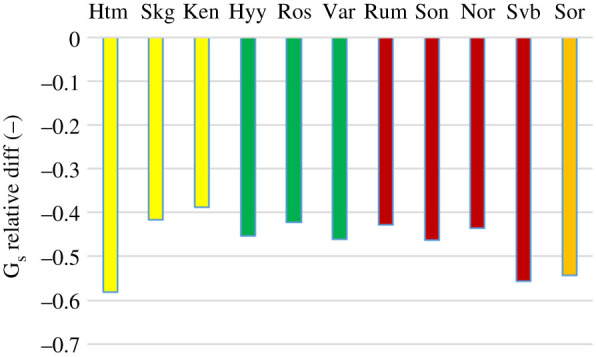
The relative difference in mean growing season surface conductance (G_s_) between year 2018 and reference year. Colour code as in figure 2. (Online version in colour.)

In contrast with the ED, the surface conductance (G_s_) was lower for all sites in 2018 as compared to the reference year, differences ranging between 39 and 58% (figure 3). Here, the variation between sites was smaller, with the lowest decrease for Kenttärova and the highest for Hyltemossa.

**Figure 4.**
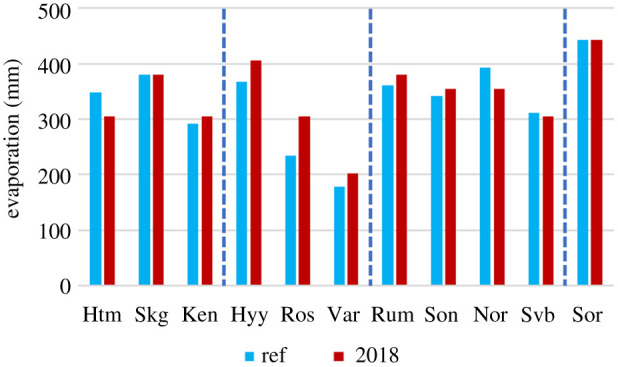
Annual evaporation for the reference year and year 2018 for all sites. The dashed vertical lines separate sites into forest types; from left to right: spruce, pine, mixed and beech, respectively. (Online version in colour.)

The increase in ED in combination with a lowering of G_s_ resulted in approximately equal or higher annual evaporation (E) for nine of the sites while Hyltemossa and Norunda showed a marked decrease in annual E (figure 4).

### (b) Carbon fluxes

**Figure 5.**
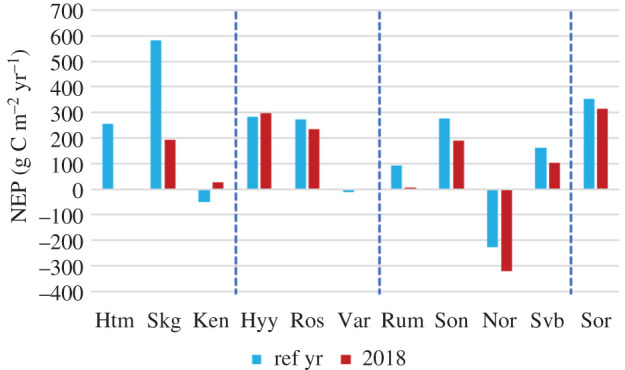
Cumulated NEP for the reference year (see electronic supplementary material Table S2) and 2018 for all sites. Note that the values that seem to be missing are zero or very close to zero fluxes. The dashed vertical lines separate sites into forest types; from left to right: spruce, pine, mixed and beech, respectively. (Online version in colour.)

The response in NEP to the 2018 weather conditions varied strongly within the studied region from strong decrease in annual NEP (e.g. the two spruce sites Hyltemossa and Skogaryd) to an increased NEP (e.g. the spruce forest Kenttärova; figure 5). Also, the annual NEP at Rumperöd, Svartberget, Soontaga and Norunda decreased during 2018, but to a much smaller extent. Several sites showed a more limited response in annual NEP to the 2018 weather conditions, i.e. the three pine sites Hyytiälä, Rosinedal and Värriö, and the beech site in Denmark. The two northernmost sites, Kenttärova and Värriö, showed increases in NEP and Kenttärova even turned from a source to a sink. Also Hyytiälä increased NEP slightly and Värriö turned from a small source to neutral. The annual values of all C-fluxes can be found in electronic supplementary material, table S2.

**Figure 6.**
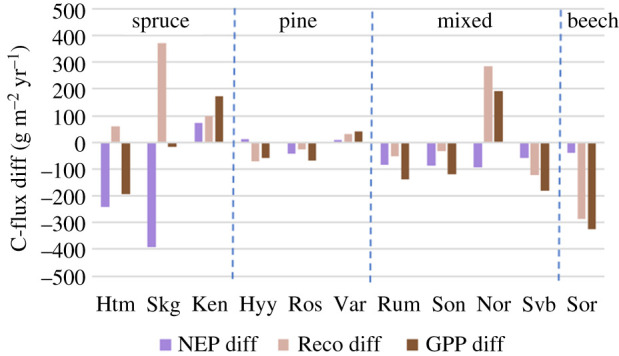
Anomalies in C-fluxes between reference year and 2018 for NEP, Reco and GPP for all sites. (Online version in colour.)

Most sites showed a decrease in annual net ecosystem productivity (purple bars with negative values in figure 6), but for different reasons; in Hyltemossa, the decrease in NEP was explained by a relatively large decrease in GPP accompanied by an increase in Reco; in Skogaryd, practically the whole decrease in NEP was explained by an increase in Reco; in Rumperöd, there was a decrease in both GPP and Reco, but the decrease in GPP was larger than in Reco; and in Sorø both GPP and Reco decreased strongly but Reco decreased slightly more resulting in the relatively small decrease in NEP (figure 6). Out of the three sites that showed increases in NEP, Kenttärova and Värriö behaved similarly by having a slightly larger increase in GPP as compared to Reco but in Hyytiälä both GPP and Reco decreased with a larger decrease in Reco (figure 6).

**Figure 7.**
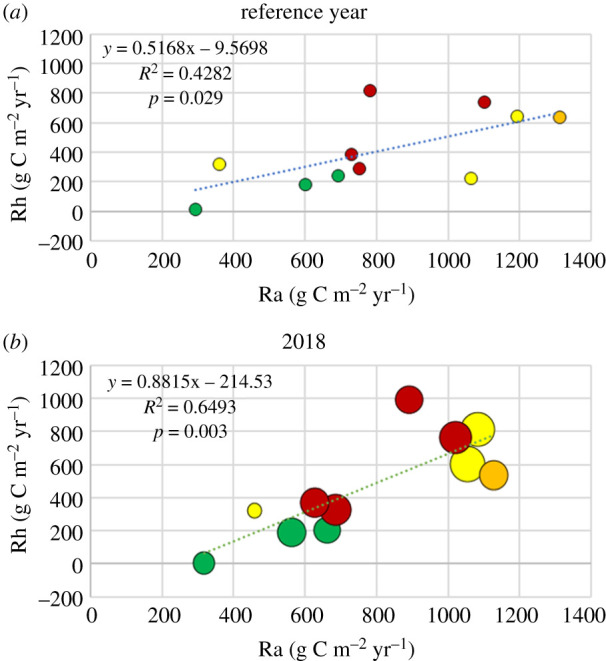
(*a*) Heterotrophic respiration (Rh) plotted against autotrophic respiration (Ra) for the respective reference year (see electronic supplementary material Table S2). (*b*) Similar plot but for the year 2018. The colour code as in figure 2. The size of the symbols for 2018 corresponds to the magnitude of the relative precipitation deficit (see also legend in figure 8). (Online version in colour.)

The heterotrophic respiration, Rh, was about 50% of the autotrophic respiration, Ra, but with quite large variation between sites during the reference year (figure 7*a*). The pine forests (green symbol) seemed to be best correlated to each other while spruce (yellow) and mixed (red) showed larger deviations from a linear relationship. The beech forest was also close to the 50% value. During the drought year, the Rh fraction of Ra increased particularly for the forests with the larger precipitation deficit (figure 7*b*).

### (c) Flux anomalies

**Figure 8.**
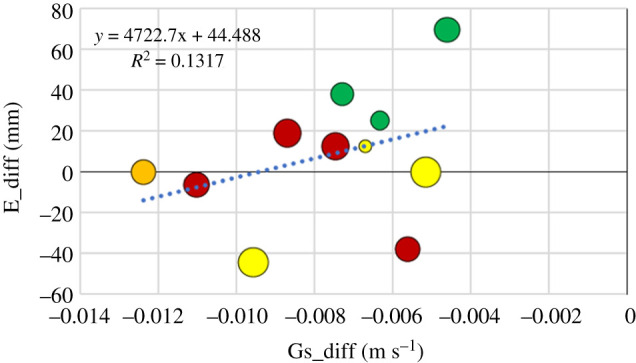
Evaporation difference (E_diff) plotted against the surface conductance difference (Gs_diff). The size of the symbols is proportional to the relative precipitation deficit, RPD (table 1). The largest symbol corresponds to RPD= 0.55 (Skogaryd) and the smallest corresponds to RPD=0.1 (Kenttärova). The colour of the symbols represents the different forest types (see legend in figure 2). (Online version in colour.)

The anomaly in evaporation, E_diff, between 2018 and reference year was poorly explained by all tested single variables. The best, but still with non-significant results, was obtained for the anomaly in surface conductance, Gs_diff (figure 8). However, combination of three independent variables, Gs_diff, RPD and GPP_diff in a multiple linear regression resulted in an increased explanation to 75% (adjusted *r*^2^; table 2).

**Table 2.** The result of the multiple linear regressions (MLR) for annual anomalies of NEP, Reco, GPP and E. Parameters RPD and Gs_diff are mean values during growing season, GPP_diff and Rh_diff are annual totals. The MLR was performed using the statistic package in Sigmaplot 12.5 [26].

dependent variable	parameter	coefficient	standard error	*p*	adjusted *r*^2^
NEP_diff (g C m^−2^ yr^−1^)	constant	134	35.2	0.005	0.934
	RPD (−)	−495	96.9	<0.001	
	Rh_diff (g C m^−2^ yr^−1^)	−0.584	0.093	<0.001	
Reco_diff (g C m^−2^ yr^−1^)	constant	−212	69.2	0.015	0.826
	RPD (−)	868	194	0.002	
	GPP_diff (g C m^−2^ yr^−1^)	1.31	0.188	<0.001	
GPP_diff (g C m^−2^ yr^−1^)	constant	474	107	0.002	0.718
	RPD (−)	−526	203	0.032	
	Gs_diff (m s^−1^)	44 060	10 672	0.003	
E_diff (mm yr^−1^)	constant	228	38.7	<0.001	0.749
	GPP_diff (g C m^−2^ yr^−1^)	−0.347	0.0690	0.002	
	Gs_diff (m s^−1^)	19 530	3685	0.001	
	RPD (−)	−243	−4.53	0.003	

**Figure 9.**
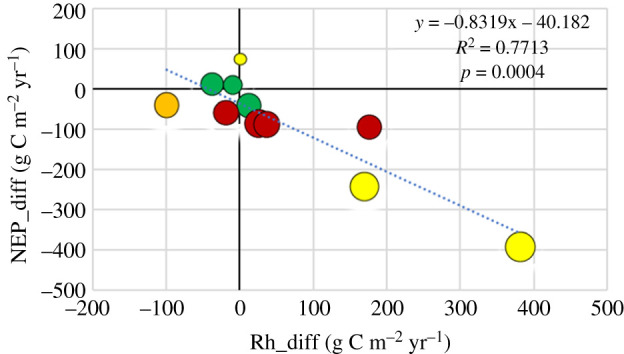
The anomaly in net primary productivity, NEP_diff, plotted against the anomaly in heterotrophic respiration, Rh_diff. Symbols as in figure 8. (Online version in colour.)

The NEP anomaly was strongly correlated with the anomaly in heterotrophic respiration, Rh_diff, with 77% of the variation explained (figure 9). Adding the relative precipitation deficit, RPD, in a multiple linear regression increased the explanation to 93% (table 2). Thus, the drought expressed as RPD did not contribute that much to the variations in NEP but still resulted in a significant improvement.

**Figure 10.**
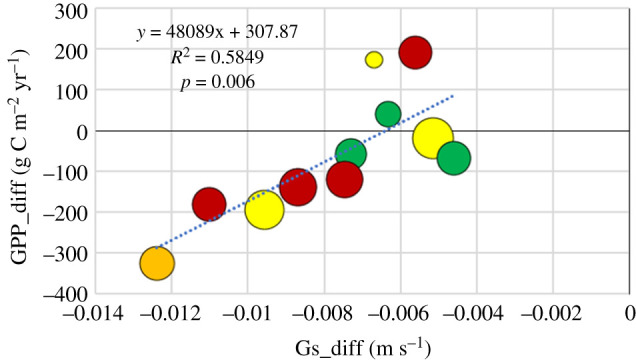
The anomaly in gross primary productivity, GPP_diff, plotted against the anomaly in surface conductance, Gs_diff. Symbols as in figure 8. (Online version in colour.)

The anomaly in gross primary productivity, GPP_diff, was reasonably well explained (58%) by the anomaly in surface conductance, Gs_diff (figure 10). Also here, adding RPD to a multiple regression increased explanation up to 71% of the variations, i.e. about the same improvement as for NEP_diff when introducing RPD in the regression.

**Figure 11.**
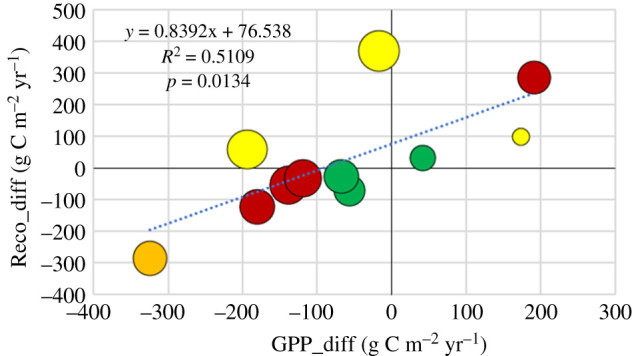
The anomaly in ecosystem respiration, Reco_diff, versus the anomaly in gross primary productivity, GPP_diff. Symbols as in figure 8. (Online version in colour.)

The anomaly in ecosystem respiration, Reco_diff, correlated well with the anomaly in GPP (figure 11). Adding RPD to the MLR regression improved the correlation significantly from 51% to 83% (table 2).

## 4. Discussion

### (a) Drought and evaporation

During 2018, the Nordic region probably experienced one of the most severe droughts for many decades, according to the SPEI drought index [21]. In North-Western Europe, the drought in 2018 was more pronounced than the one in 2003. The drought was especially severe in Denmark, the southern part of Sweden and Estonia (figure 1 and electronic supplementary material, figure S1) where five of the studied sites were located: Sorø, Hyltemossa, Rumperöd, Skogaryd and Soontaga. All of these sites had at least a 3-month-long period of severe drought during the early part and peak of the growing season (May–July) while the other sites had a recovery in June with mild or no drought during that month (electronic supplementary material. figure S1). Soontaga was exceptional with drought throughout the whole year, while Norunda experienced the strongest drought for a single month with an SPEI index of −4.3 in May.

Unexpectedly, the drought reduced annual E at only two of the sites: Hyltemossa and Norunda (figure 4). At all other sites, annual E was higher than or equal to the reference year. This occurred despite a strong reduction of surface conductance at all sites (figure 3). Surface conductance is known to constitute an important control of transpiration rate but, in a drought situation, increased evaporative demand counteracts this by increasing the evaporation rate per unit stomatal conductance due to higher surface heating and high vapour pressure deficit, all of which are acting to enhance evaporation. This is reflected in the observed increases in evaporative demand (figure 2) which thus contributed to reducing the negative impact of drought on evaporation.

The results show that the majority of forest could cope well with the drought and maintain close to normal or even higher evaporation rates. Pine stands tended to reduce Gs less and thus increased E in extreme drought, an indicator of a more wasteful pattern of water use.

Many studies have shown that surface conductance and transpiration become reduced when soil moisture drops below some threshold (e.g. [27,28]). However, total evaporation is constituted not only by tree transpiration but also transpiration from forest floor vegetation (if present), interception evaporation and soil evaporation. Drought can affect these components differently, although in forests with closed canopies, the transpiration is normally the largest component. During extensive drought, it is still likely that transpiration is dominating the total evaporation since there is no or little rain and thus no intercepted evaporation and soil evaporation is most likely also diminished because of the drying topsoil.

For species with shallow root systems such as spruce, hydraulic lift of water from deeper horizons below the rooting zone [29,30] could also be part of the explanation to the sustained evaporation rates. It has also been suggested that changes in carbon allocation patterns due to the drought could stimulate root growth into deeper soil layers. Mackay *et al*. [31] tested this hypothesis for conifer trees using a new modelling framework, and they concluded that the trees shifted water uptake among existing roots rather than growing new roots. Oishi *et al*. [32], who studied water relations of an oak-hickory forest in North Caroline, USA, for several years including 1 year with severe drought, also found that the canopy transpiration was not reduced by drought for the same reason

For reasons discussed above, it was not entirely unexpected that the Gs anomaly did not explain the anomaly in evaporation, E_diff (figure 8). Even adding more factors, like the relative precipitation deficit only, or the anomaly in evaporative demand, ED_diff, to the regression did not improve the results. This is an indication for the stands being able to effectively maintain the water flows, even in largely variable weather situations. Interestingly, when the GPP anomaly was added, the degree of explanation increased significantly (table 2). One explanation is that stomatal regulation is intricately linked with photosynthesis, as the overwhelming empirical evidence at leaf scale shows. Empirical stomatal conductance models make Gs dependent on photosynthesis, corrected for the effects on the leaf of air water vapour concentration difference, acting to stabilize water use efficiencies. The correlation can thus be both ways, where cause and effect cannot be distinguished. In any case, our results confirm the strong interrelation between Gs and GPP. Another possibility could be that the GPP anomaly better reflects the whole year than the Gs and ED anomalies which only represent the growing season and dry conditions. This must be of particular importance for temperature-limited sites, where the warmer climate even increases GPP.

### (b) Drought effects on carbon fluxes

The drought in 2003 had a dramatic impact on the European carbon balance resulting in a net source of 0.5 Pg yr^−1^ of carbon into the atmosphere, counterbalancing four years of carbon sink [13]. The range in NEP anomalies among the twelve forests which were part of that study and which reported annual numbers was −155 to +366 g C m^−2^ yr^−1^ with a median value of −43 g C m^−2^ yr^−1^. The corresponding values in our study were NEP anomalies in the range of −389 to +74 g C m^−2^ yr^−1^ with a median value of −59 g C m^−2^ yr^−1^. Thus, the variation between sites was of the same magnitude but the 2018 drought had an even greater negative impact on NEP compared to the 2003 extreme drought.

We did not expect to find a consistent explanation for the changes in NEP between sites but rather for the changes in the underlying processes, GPP and Reco. The NEP is then rather to be understood as consequential. Three of the sites that showed a relatively large negative impact on NEP (Hyltemossa, Skogaryd and Norunda), showed an increase in Reco, but of very different magnitude (figure 6), while five sites (Rosinedal, Rumperöd, Soontaga, Svartberget and Sorø) showed a decrease in Reco. The response of Reco to drought conditions is complex because Reco is composed of fluxes with very different individual responses, i.e. heterotrophic respiration and autotrophic respiration (the latter including aboveground canopy and belowground root components). The relative contribution from autotrophic and heterotrophic respiration to soil respiration may vary substantially but is commonly about 50% each in forest ecosystems (e.g. [14]). The vast majority of the aboveground respiration represents autotrophic respiration, thus it is very likely that the majority of Reco represents autotrophic respiration. It is also well documented that GPP has a major influence on Reco [33] in total as well as soil respiration specifically [34]. This strong linkage between Reco and GPP is well illustrated by our result showing that about half of the anomaly in Reco is explained by the corresponding anomaly in GPP (figure 11). We also demonstrate that the drought, expressed as the relative precipitation deficit during the growing season, explained additionally 30% of this anomaly.

An increase in Reco due to warmer weather is according to expectations, but soil moisture and oxygen level in the soil are also affecting Reco through their effects on soil respiration. We observed a shift in the relationship between Rh and Ra towards relatively higher Rh during 2018 compared to the reference year (figure 7). We attribute this shift to an effect of the drought, resulting in a lowering of Ra and an increase of Rh at most of the sites (electronic supplementary material, table S2). Sierra *et al*. [35] conducted an incubation experiment using a boreal soil demonstrating how the combination of temperature, soil moisture and oxygen level affected respiration rates. Low soil moisture generally decreased respiration, more at low oxygen levels than at high, but the temperature sensitivity was always maintained. It is thus the balance between these factors that determine whether the respiration will increase or decrease for certain conditions in soil and weather.

Litton *et al*. [25] analysed productivity and respiration from a large range of diverse forest ecosystems and for 23 of these they could estimate the relationship between GPP and autotrophic respiration, Ra. The relationship Ra = 0.57 x GPP explained 95% (*p* < 0.01) of the variation. We used this relationship to estimate Ra in our study and it turned out that the NEP anomaly was largely explained by the anomaly in Rh (figure 9). Rh alone explained 77% of the variation in NEP and adding relative precipitation deficit to the regression increased explanation to 93% (table 2). However, it should be pointed out that there was indeed some variation between sites in the Litton *et al*. [25] study with a range of 0.42 to 0.71 for the fraction of GPP partitioned to Ra. Wu *et al.* [36] determined a slightly but insignificantly higher value of 0.62 ± 0.08 for the Sorø beech forest over 5 years. We also plotted the Rh values on a daily scale (electronic supplementary material, figure S2*a*–*f*) but these values should be interpreted with care since the relationship used was developed for annual values and, thus, does not represent possible seasonal variation in relationship between Ra and GPP.

There were large variations in GPP anomalies between sites with a range of −325 g C m^−2^ (Sorø) to +191 g C m^−2^ (Norunda) (figure 6). Out of the sites that showed a decrease in NEP, Norunda was the only site that showed an increase in GPP. All others showed a decrease, from very minor (Skogaryd; −17.5 g C m^−2^) to quite large (Sorø; −325 g C m^−2^). The anomaly in GPP was expected to be well explained by the anomaly in surface conductance, Gs_diff (figure 11), since surface conductance is an integrated measure of stomatal conductance, and stomatal conductance and photosynthesis are mechanistically linked (e.g. [37,38]). However, the explained variation in GPP was not more than 58% and adding the relative precipitation deficit to the multilinear model only improved the explanatory degree by 14% (table 2). As shown by Gourlez de la Motte [39], the enzymatic processes of carboxylation can also be directly downregulated if the relative extractable water falls below a critical threshold; this would explain a direct role of water deficits for the GPP. An additional reason could be that our estimate of surface conductance was made only for dry daytime conditions during the growing season and that the shoulder seasons, which are included in the GPP anomaly, are influencing this relationship in a different way.

The most common type of response in our study was a decrease in both GPP and Reco, with a smaller decrease in the latter resulting in a decrease in NEP. Such responses are in line with those found in the European drought study [13] as well as in a recent study on North American forest ecosystems [9]. In the latter study, they concluded that in coniferous forests drought had a similar dampening effect on both Reco and GPP, therefore resulting in a neutral impact on NEP, while in deciduous forests, the decrease in NEP was mostly driven by a decrease in GPP. We do not find such clear distinction in responses between forest types but it should be pointed out that many of the forests in this study were mixed conifer/deciduous trees (electronic supplementary material, table S2).

## 5. Conclusion

We conclude that the drought in 2018 was severe across the Nordic region and much more distinct than the drought in 2003. We found that despite the drought being the most severe over the last five decades (according to the SPEI) the effect on annual NEP was not always drastic and instead varied considerably between the 11 forests included in the study. However, it should be pointed out that eight out of the 11 forests still acted as annual C sinks. In six out of 11 forests, the annual net ecosystem productivity was reduced by more than 50 g C m^−2^ yr^−1^. In one forest, the net uptake was reduced by as much as 389 g C m^−2^ yr^−1^. But we also found that two of the forests in the north benefited from the favourable weather conditions (i.e. increased Tair) during the drought and actually increased their net uptake. The main conclusions regarding the drivers of C-flux anomalies during drought are:

— 77% of the NEP anomaly is explained by the anomaly in heterotrophic respiration, Rh, and 16% by the relative precipitation deficit during the growing season.
— 51% of the Reco anomaly is likewise explained by GPP anomaly and 31% by the relative precipitation deficit.
— 58% of the GPP anomaly is explained by the anomaly in surface conductance during the growing season and 14% by the relative precipitation deficit.

Evaporation actually stayed the same or even increased at the majority of sites in spite of the drought and in spite of the strong reductions in surface conductance. The evaporation anomaly could only be explained by a linear combination of three variables: the surface conductance anomaly, the relative precipitation deficit and the GPP anomaly. Together these three variables explained 75% of the variation in the evaporation anomaly.

## Supplementary Material

Supplement to: Effects of drought and meteorological forcing on carbon and water fluxes in Nordic forests during the dry summer of 2018

## Data accessibility

The data will be published at the ICOS Carbon Portal.

## References

[1] Friedlingstein P et al. 2019 Global carbon budget 2019. Earth Syst. Sci. Data 11, 1783–1838. (10.5194/essd-11-1783-2019)

[2] Frank D et al. 2015 Effects of climate extremes on the terrestrial carbon cycle: concepts, processes and potential future impacts. Glob. Change Biol. 21, 2861–2880. (10.1111/gcb.12916) PMC4676934 25752680

[3] Litell JS, Peterson DL, Riley KL, Liu Y, Luce CH 2016 A review of the relationships between drought and forest fire in the United States. Glob. Change Biol. 22, 2353–2369. (10.1111/gcb.13275) 27090489

[4] Schlesinger WH, Dietze MC, Jackson RB, Phillips RP, Rhoades CC, Rustad LE, Vose JM 2016 Forest biogeochemistry in response to drought. Glob. Change Biol. 22, 2318–2328. (10.1111/gcb.13105) 26403995

[5] Xiao J, Liu S, Stoy PC 2016 Preface: impacts of extreme events and disturbances on carbon dynamics. Biogeosciences 13, 3665–3675. (10.5194/bg-13-3665-2016)

[6] IPCC. 2013 Climate change 2013: the physical science basis. In Contribution of working group I to the fifth assessment report of the intergovernmental panel on climate change (eds Stocker TF et al), pp. 1535 Cambridge, UK: Cambridge University Press.

[7] Kljun N, Black TA, Griffis TJ, Barr AG, Gaumont-Guay D, Morgenstern K, McCaughey JH, Nesic Z 2006 Response of net ecosystem productivity of three boreal forest stands to drought. Ecosystems 9, 1128–1144. (10.1007/s10021-007-9088-x)

[8] Welp LR, Randerson JT, Liu HP 2007 The sensitivity of carbon fluxes to spring warming and summer drought depends on plant functional type in boreal ecosystems. Agric. For. Meteorol. 147, 172–185. (10.1016/j.agrformet.2007.07.010)

[9] Xu B, Arain MA, Black TA, Law BE, Pastorello GZ, Chu H 2019 Seasonal variability of forest sensitivity to heat and drought stresses: a synthesis based on carbon fluxes from North American forest ecosystems. Glob. Change Biol. 26, 901–918. (10.1111/gcb.14843) 31529736

[10] Wolf S et al. 2016 Warm spring reduced carbon cycle impact of the 2012 US summer drought. Proc. Natl Acad. Sci. USA 113, 5880–5885. (10.1073/pnas.1519620113) 27114518 PMC4889356

[11] Angert A, Biraud S, Bonfils C, Henning CC, Buermann W, Pinzon J, Tucker CJ, Fung I, Field CB 2005 Drier summers cancel out the CO_2_ uptake enhancement induced by warm springs. Proc. Natl Acad. Sci. USA 102, 10 823–10 827. (10.1073/pnas.0501647102) PMC1180508 16043702

[12] Pan Y, Schimel D 2016 Synergy of a warm spring and dry summer. Nature 534, 483–484. (10.1038/nature18450) 27309806

[13] Ciais P et al. 2005 Europe-wide reduction in primary productivity caused by the heat and drought in 2003. Nature 437, 529–533. (10.1038/nature03972) 16177786

[14] Högberg P, Nordgren A, Buchmann N, Taylor AFS, Ekblad A, Högberg MN, Nyberg G, Ottosson-Löfvenius M, Read DJ 2001 Large-scale forest girdling shows that current photosynthesis drives soil respiration. Nature 411, 789–792 (10.1038/35081058). 11459055

[15] Reichstein M et al. 2007 Reduction of ecosystem productivity and respiration during the European summer 2003 climate anomaly: a joint flux tower, remote sensing and modelling analysis. Glob. Change Biol. 13, 634–651. (10.1111/j.1365-2486.2006.01224.x)

[16] Von Butlar J et al. 2018 Impacts of droughts and extreme-temperature events on gross primary production and ecosystem respiration: a systematic assessment across ecosystems and climate zones. Biogeosciences 15, 1293–1318. (10.5194/bg-15-1293-2018)

[17] Noormets A, McNulty SG, DeForest JL, Sun G, Li Q, Chen J 2008 Drought during canopy development has lasting effect on annual carbon balance in a deciduous temperate forest. New Phytol. 179, 818–828. (10.1111/j.1469-8137.2008.02501.x) 18537894

[18] Grant RF, Barr AG, Black TA, Margolis HA, Dunn AL, Metsarana J, Wang S, McCaughey JH, Borque CA 2009 Interannual variation in net primary productivity of Canadian forests as affected by regional weather patterns—a Fluxnet-Canada synthesis. Agric. For. Meteorol. 149, 2022–2039. (10.1016/j.agrformet.2009.07.010)

[19] Ma Z, Peng C, Zhu Q, Chen H, Yu G, Li W, Zhou X, Wang W, Zhang W 2012 Regional drought-induced reduction in biomass carbon sink of Canada's boreal forests. Proc. Natl Acad. Sci. USA 109, 2423–2427. (10.1073/pnas.1111576109) 22308340 PMC3289349

[20] Walker XJ, Mack MC, Johnstone JF 2015 Stable carbon isotope analysis reveals widespread drought stress in boreal black spruce forests. Glob. Change Biol. 21, 3102–3113. (10.1111/gcb.12893) 25683740

[21] Vicente-Serrano SM, Beguería S, López-Moreno JL 2010 A Multi-scalar drought index sensitive to global warming: the Standardized Precipitation Evapotranspiration Index—SPEI. J. Clim. 23, 1696–1718. (10.1175/2009JCLI2909.1)

[22] Wutzler T, Lucas-Moffat A, Migliavacca M, Knauer J, Sickel K, Šigut L, Menzer O, Reichstein M 2018 Basic and extensible post-processing of eddy covariance flux data with REddyProc. Biogeosciences 15, 5015–5030. (10.5194/bg-15-5015-2018)

[23] Monteith JL 1965 Evaporation and atmosphere. In The state and movement of water in living organisms; 19th Symp. Soc. Exp. Biol. (ed. Fogg GE), pp. 206–234. Cambridge, UK: The Company of Biologists.

[24] Monteith JL, Unsworth M 2008 Principles of environmental physics, 3rd edn London, UK: Academic Press.

[25] Litton CM, Raich JW, Ryan MG 2007 Carbon allocation in forest ecosystems. Glob. Change Biol. 13, 2089–2109. (10.1111/j.1365-2486.2007.01420.x)

[26] Systat Software. 2003 *SigmaPlot 12.5 User's Guide*. San Jose, CA: Systat Software.

[27] Irvine J, Perks MP, Magnani F, Grace J 1998 The response of *Pinus sylvestris* to drought: stomatal control of transpiration and hydraulic conductance. Tree Physiol. 18, 393–402. 12651364 10.1093/treephys/18.6.393

[28] Lagergren F, Lindroth A 2002 Transpiration response to soil moisture in pine and spruce trees in Sweden. Agric. For. Meteorol. 112, 67–85. (10.1016/S0168-1923(02)00060-6)

[29] Emerson SH, Dawson TE 1996 Hydraulic lift and its influence on the water content of the rhizosphere: an example from sugar maple, *Acer saccarum*. Oecologia 108, 273–278. 28307839 10.1007/BF00334651

[30] Caldwell MM, Dawson TE, Richards JH 1998 Hydraulic lift: consequences of water efflux from the roots of plants. Oecologia 113, 151–161. 28308192 10.1007/s004420050363

[31] Mackay DS, Savoy PR, Grossiord C, Tai X, Pleban J, Wang DR, McDowell NG, Adams HD, Sperry JS 2020 Conifers depend on established roots during drought: results from a coupled model of carbon allocation and hydraulics. New Phytologist 225, 679–692. (10.1111/nph.16043) 31276231

[32] Oishi AC, Oren R, Novick KA, Palmroth S, Katul GG 2010 Interannual variability of forest evapotranspiration and its consequences to water flow downstream. Ecosystems 13, 421–436. (10.1007/s10021-010-9328-3)

[33] Larsen KS, Ibrom A, Beier C, Jonasson S, Michelsen A 2007 Ecosystem respiration depends strongly on photosynthesis in a temperate heath. Biogeochemistry 85, 201–213. (10.1007/s10533-007-9129-8)

[34] Janssens IA et al. 2001 Productivity overshadows temperature in determining soil and ecosystem respiration across European forests. Glob. Change Biol. 7, 269–278 (10.1046/j.1365-2486.2001.00412.x).

[35] Sierra CA, Malghani S, Loescher HW 2017 Interactions among temperature, moisture, and oxygen concentrations in controlling decomposition rates in a boreal forest soil. Biogeosciences 14, 703–710. (10.5194/bg-14-703-2017)

[36] Wu J, Larsen KS, van der Linden L, Beier C, Pilegaard K, Ibrom A 2013 Synthesis on the carbon budget and cycling in Danish temperate deciduous forest. Agric. For. Meteorol. 181, 94–107. (10.1016/j.agrformet.2013.07.012)

[37] Whitehead D 1997 Regulation of stomatal conductance and transpiration in forest canopies. Tree Physiol. 18, 633–644. (10.1093/treephys/18.8-9.633) 12651352

[38] Farquhar GD, von Caemmerer S 1982 Modelling of photosynthetic response to environmental conditions. In Physiological plant ecology II. Encyclopedia of plant physiology (New series), vol. 12 / B (eds Lange OL, Nobel PS, Osmond CB, Ziegler H). Berlin, Germany: Springer.

[39] Gourlez de la Motte L et al 2020 Non-stomatal processes reduce gross primary productivity in temperate forest ecosystems during severe edaphic drought. Phil. Trans. R. Soc. B 375, 20190527 (10.1098/rstb.2019.0527) 32892725 PMC7485095

