# Supplementary material for: Effects of drought and meteorological forcing on carbon and water fluxes in Nordic forests during the dry summer of 2018
Source: Philos Trans R Soc Lond B Biol Sci. 2020 Sep 7;375(1810):20190516. doi: 10.1098/rstb.2019.0516 (PMC7485108; doi:10.1098/rstb.2019.0516)
Supplement: Supplement to: Effects of drought and meteorological forcing on carbon and water fluxes in Nordic forests during the dry summer of 2018 [file rstb20190516supp1.pdf]

Supplement to: Effects of drought and meteorological forcing on carbon and water fluxes in Nordic forests during the dry summer of 2018

Anders Lindroth[1], Jutta Holst[1], Maj-Lena Linderson[1], Mika Aurela[2], Tobias Biermann[3], Michal Heliasz[3], Jinshu Chi[4], Andreas Ibrom[5], Pasi Kolari[6], Leif Klemedtsson[7], Alisa Krasnova[8], Tuomas Laurila[2], Irene Lehner[3], Annalea Lohila[2], Ivan Mammarella[6], Meelis Mölder[1], Mikaell Ottosson Löfvenius[4], Matthias Peichl[4], Kim Pilegaard[5], Kaido Soosaar[8], Timo Vesala[6], Patrik Vestin[1], Per Weslien[7] and Mats Nilsson[4].

[1] Department of Phycical Geography and Ecosystem Science, Lund University, Lund, Sweden
[2] Finnish Meteorological Institute, Helsinki, Finland
[3] Centre for Environmental and Climate Research, Lund University, Lund, Sweden
[4] Department of Forest Ecology and Management, the Swedish University of Agricultural Sciences, Umeå, Sweden
[5] Department of Environmental Engineering, Technical University of Denmark, Lyngby, Denmark
[6] Institute for Atmospheric and Earth System Research, Helsinki University, Helsinki, Finland
[7] Department of Earth Sciences, University of Gothenburg, Gothenburg, Sweden
[8] Institute of Ecology and Earth Sciences, University of Tartu, Tartu, Estonia

The 30-year mean monthly air temperature and the monthly air temperature during 2018 from nearest weather station (see Tables S1 and S2) operated by the respective countries meteorology service are shown in Fig. S1. Also shown is the monthly 2008 SPEI drought index obtained from the Global Drought Monitor (https://spei.csic.es/map/maps.html). This index uses the period 1950 – 2010 as a reference and the spatial resolution is 1°. More information about SPEI can be found in Vicente-Serrano et al. (2010).

Table S1. Name of weather station nearest to the respective flux station.

Name of flux station	Name of weather station/country
Hyltemossa (Htm)	Barkåkra_Klippan/Sweden
Hyytiälä (Hyy)	Juupanjokki_Hyytiälä/Finland
Kenttärova (Ken)	Karesuando/Sweden
Norunda (Nor)	Vattholma_Uppsala/Sweden
Rosinedal (Ros)	Hällnäs_Lund/Sweden
Rumperöd (Rum)	Osby/Sweden
Skogaryd (Skg)	Kroppefjäll_Granan/Sweden
Soontaga (Son)	Valga/Estonia
Sorø (Sor)	Copenhagen_Landbo/Denmark
Svartberget (Svb)	Hällnäs_Lund/Sweden
Värriö (Var)	Salla_Värriötunturi/Finland

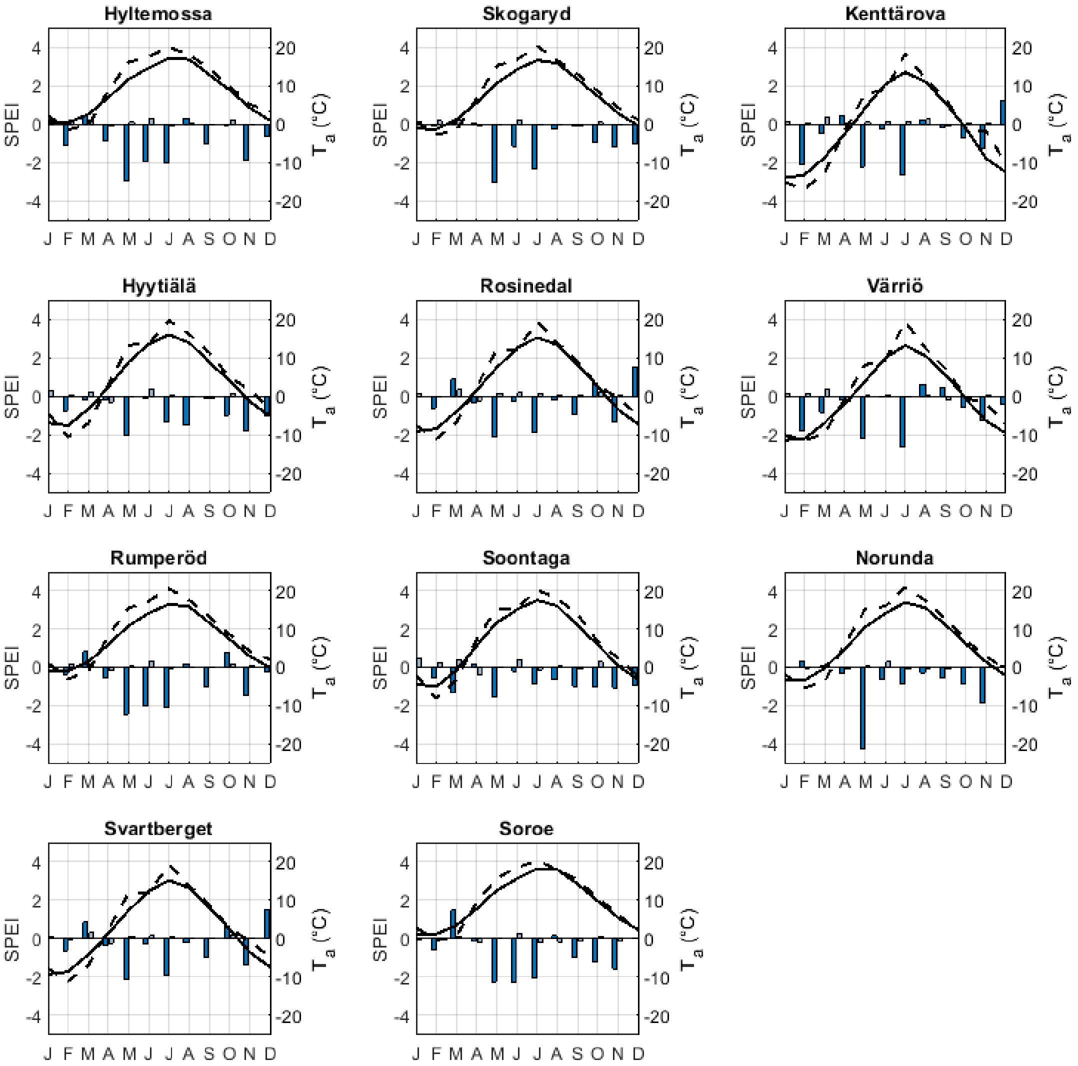

Figure S1. Monthly air temperature during 2018 compared to the long term mean climate for all measurement stations and SPEI drought index for 2018 and for the normal period 1981-2010. Blue bars correspond to the 1-month SPEI index 2018 and open bars to the normal values (Vicente-Serrano et al., 2010). Solid line is 1981-2010 monthly air temperature and hatched line is monthly air temperature 2018. Temperature data origins from weather services in Denmark, Sweden, Finland and Estonia.

Annual mean values (or cumulated sums) of NEP, Reco, GPP, Ra, Rh and air temperature for the selected reference year and for 2018 are presented in Table S2. Also shown are climatological mean values from nearby weather stations. The selected reference year (single year or mean of two years) are also presented in Table S2.

Seasonal variation of respiration components (Reco, Ra and Rh), soil moisture and air temperature for reference years and for 2018 are shown in Fig. S2a-f.

Table S2. Annual C-fluxes from the different sites, for reference year and for 2018. From nearby weather stations comes mean annual air temperature and precipitation for reference year and 2018 (Ta and P) as well as for the period 1981-2010 (Ta_c and P_c).

Site specific data								From nearby weather stations			
Site	Year	NEP	Reco	GPP	Ra	Rh	E	Ta	P	Ta_c	P_c
		g C/m2	g C/m2	g C/m2	g C/m2	g C/m2	mm	°C	mm	°C	mm
Htm	2015/2017	256.4	1836.4	2092.8	1192.9	643.5	348.0	8.9	895.7	8.1	792.1
	2018	3.6	1895.4	1899.0	1082.5	812.9	304.0	9.4	462.0		
	Diff	-252.7	58.9	-193.8	-110.5	169.4	-44.0	*0.6*	*-433.7*		
Skg	2015	582.3	1286.8	1869.0	1065.3	221.4	380.0	8.2	875.0	6.8	802.6
	2018	192.9	1658.7	1851.6	1055.4	603.3	380.0	8.1	599.2		
	Diff	-389.4	371.9	-17.5	-10.0	381.9	0.0	*-0.1*	*-275.8*		
Ken	2010/2011	-48.6	681.0	632.4	360.5	320.5	291.0	-1.9	579.4	-1	522.1
	2018	25.3	780.6	806.0	459.4	321.3	304.0	0.0	360.8		
	Diff	73.9	99.7	173.5	98.9	0.8	13.0	*1.9*	*-218.6*		
Hyy	2015/2016	283.6	933.6	1217.2	693.8	239.8	367.0	4.9	659.0	3.5	712
	2018	296.5	863.6	1160.2	661.3	202.4	405.0	4.8	540.0		
	Diff	12.9	-70.0	-57.1	-32.5	-37.4	38.0	*0.0*	*-119.0*		
Ros	2015/2017	274.7	780.2	1054.9	601.3	178.9	234.0	3.0	574.8	2.3	572.8
	2018	233.5	753.6	987.1	562.7	191.0	304.0	3.4	432.9		
	Diff	-41.2	-26.6	-67.8	-38.6	12.0	70.0	*0.5*	*-141.9*		
Var	2016	-13.4	529.3	515.9	294.0	13.4	177.0	1.3	660.4	-0.5	601.7
	2018	-3.6	560.9	557.2	317.6	3.6	203.0	1.2	505.1		
	Diff	9.8	31.6	41.4	23.6	-9.8	25.0	*-0.1*	*-155.3*		
Rum	2015/2017	92.4	1838.5	1930.9	1100.6	737.9	361.0	8.1	673.2	7.1	772
	2018	6.9	1785.4	1792.3	1021.6	763.8	380.0	8.7	529.4		
	Diff	-85.4	-53.2	-138.6	-79.0	25.8	19.0	*0.6*	*-143.8*		
Son	2016/2017	276.5	1043.8	1320.3	752.6	291.2	342.0	6.4	757.9	5.9	701
	2018	188.8	1012.5	1201.3	684.7	327.8	355.0	7.0	511.0		
	Diff	-87.7	-31.3	-119.1	-67.9	36.5	13.0	*0.6*	*-246.9*		
Nor	2015	-226.3	1598.9	1372.6	782.4	816.5	393.0	7.2	592.0	6	621.4
	2018	-320.8	1884.1	1563.3	891.1	993.1	355.0	7.3	449.1		
	Diff	-94.6	285.2	190.7	108.7	176.5	-38.0	*0.1*	*-142.9*		
Svb	2016	163.3	1118.5	1281.8	730.6	387.8	310.0	3.1	476.1	2.3	572.8
	2018	104.5	996.7	1101.1	627.7	369.0	304.0	3.4	432.9		
	Diff	-58.9	-121.8	-180.6	-103.0	-18.8	-6.0	*0.3*	*-43.2*		
Sor	2015/2016	353.8	1948.6	2302.4	1312.3	636.2	443.0	10.1	668.8	9.2	851
	2018	313.6	1663.8	1977.4	1127.1	536.7	443.0	10.0	493.0		
	Diff	-40.2	-284.8	-324.9	-185.2	-99.6	0.0	*-0.1*	*-175.8*		

Hyltemossa

Skogaryd

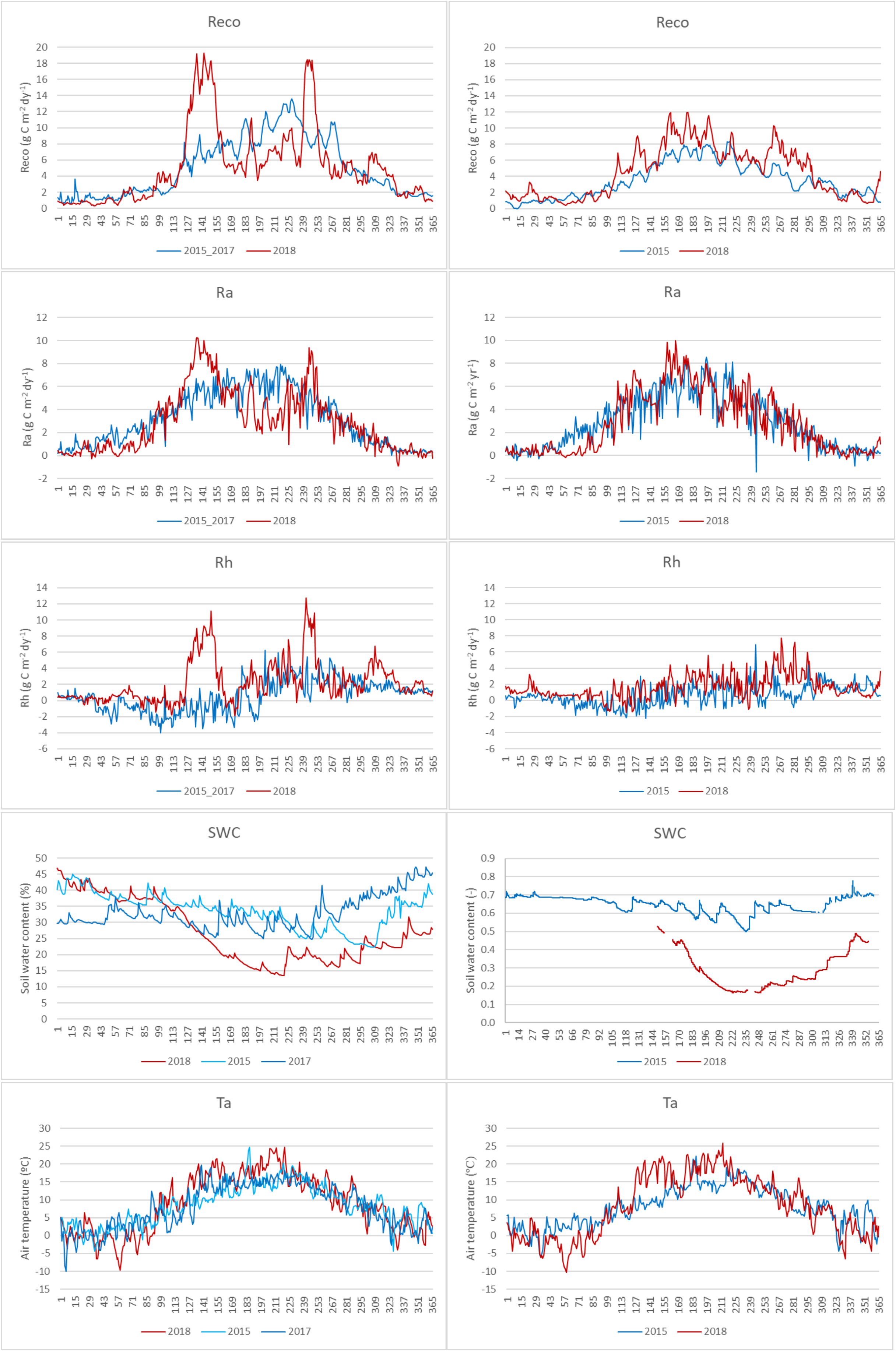

Figure S2a. Seasonal variation of respiration components, soil moisture and air temperature at Hyltemossa and Skogaryd. Soil moisture in Skogaryd from adjacent site with same type of forest but on organic soil.

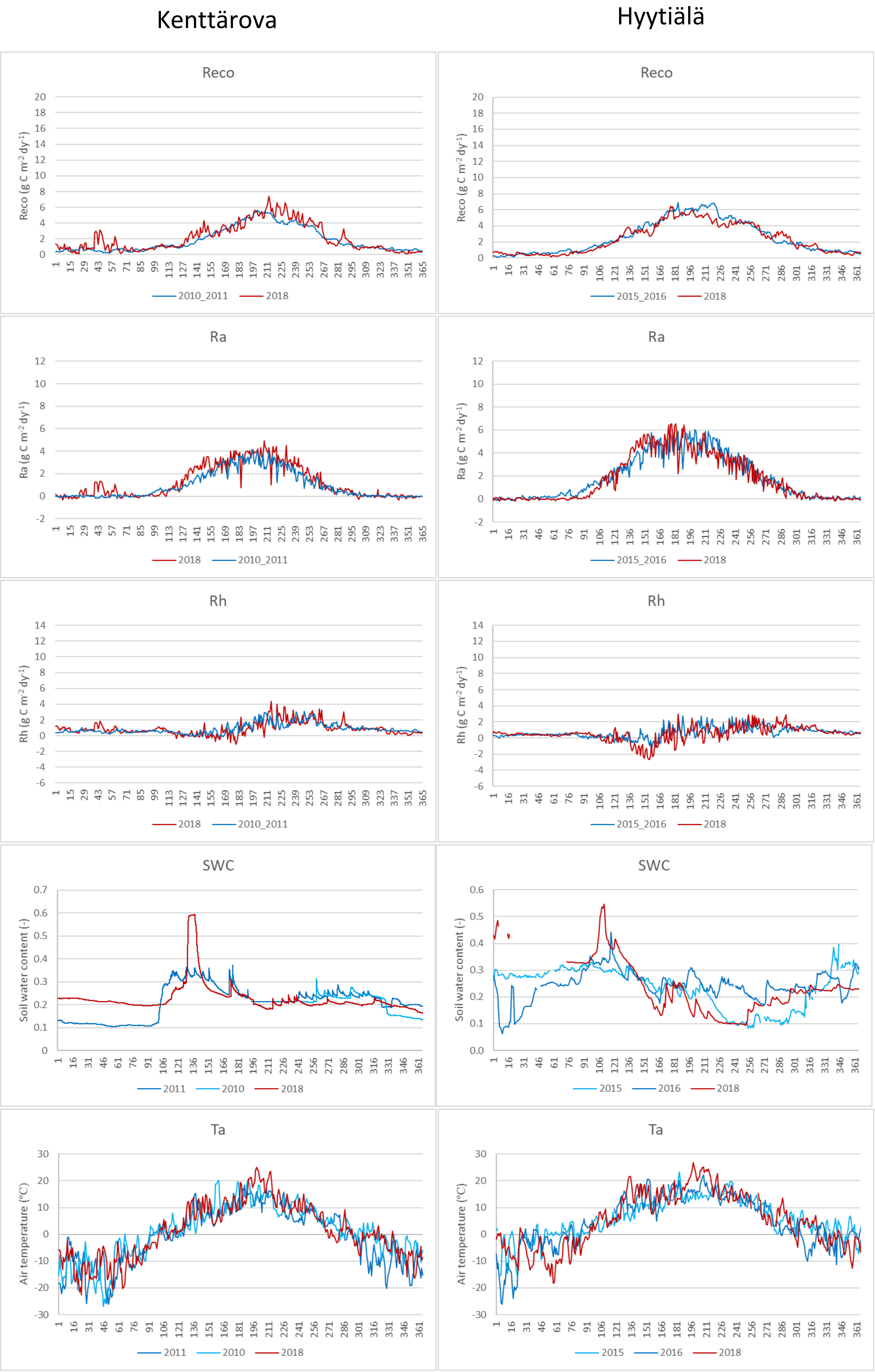

Figure S2b. Seasonal variation of respiration components, soil moisture and air temperature at Kenttärova and Hyytiälä.

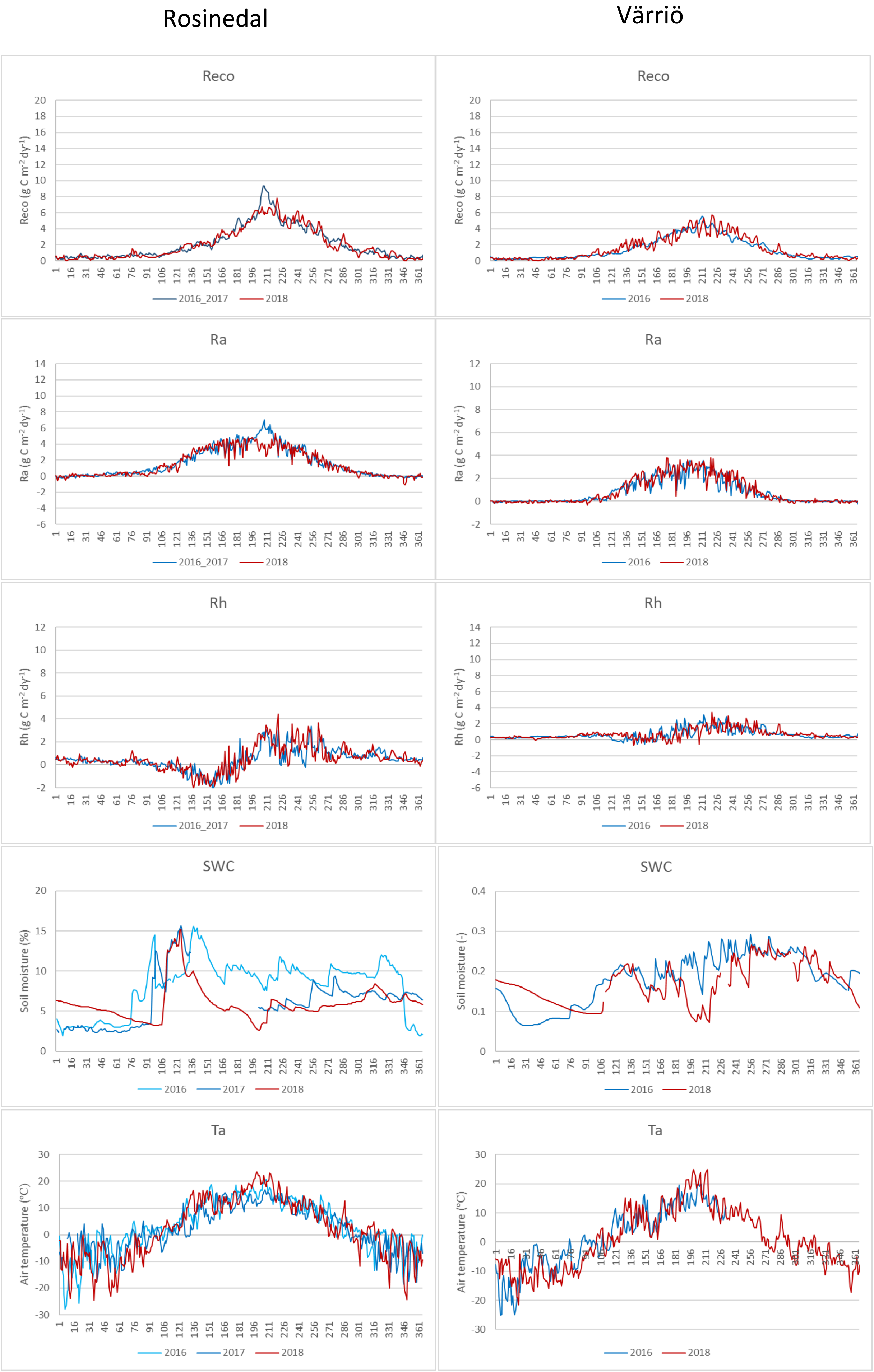

Figure S2c. Seasonal variation of respiration components, soil moisture and air temperature at Rosinedal and Värriö.

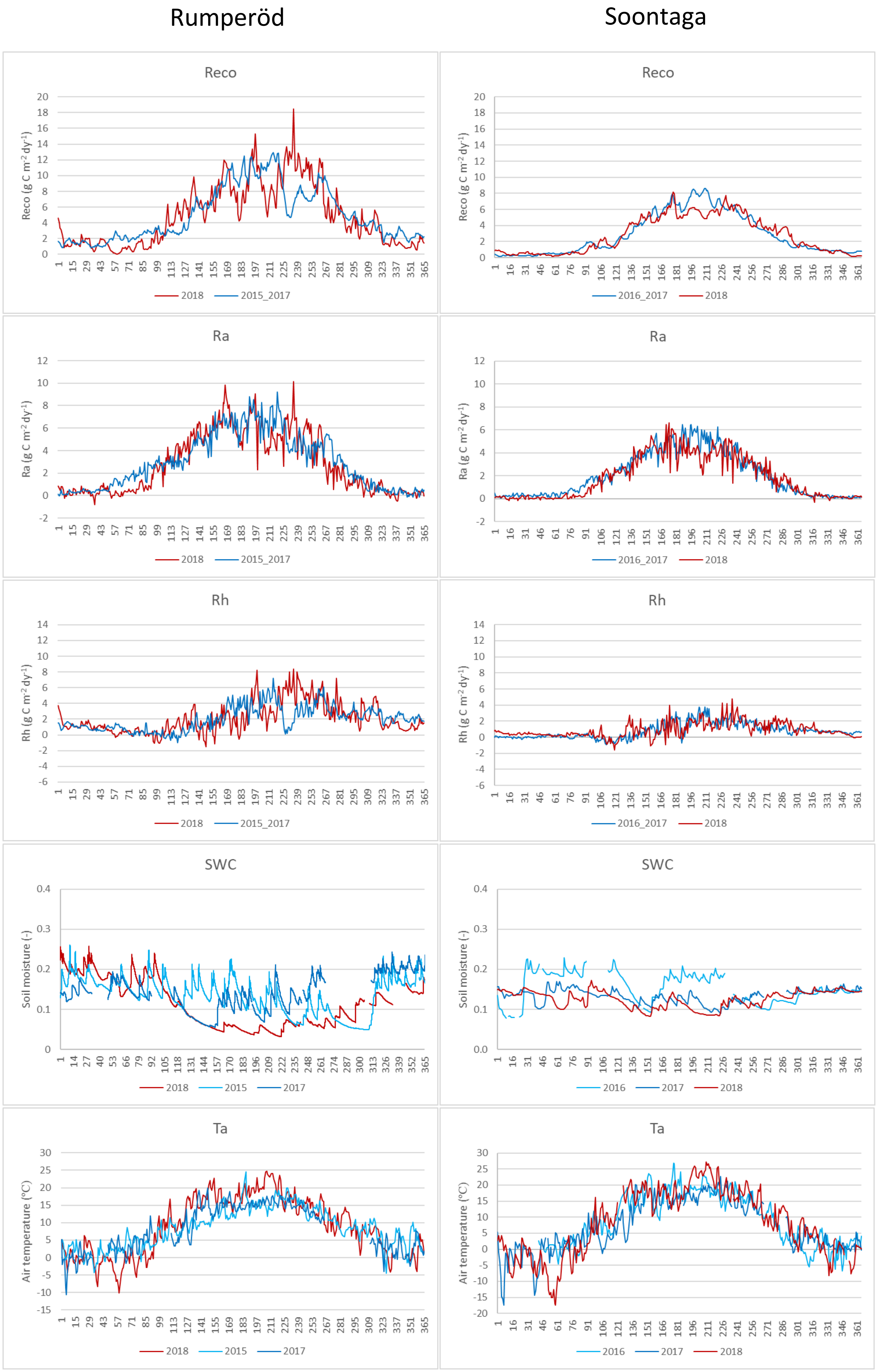

Figure S2d. Seasonal variation of respiration components, soil moisture and air temperature at Rumperöd and Soontaga.

Norunda | Svartberget

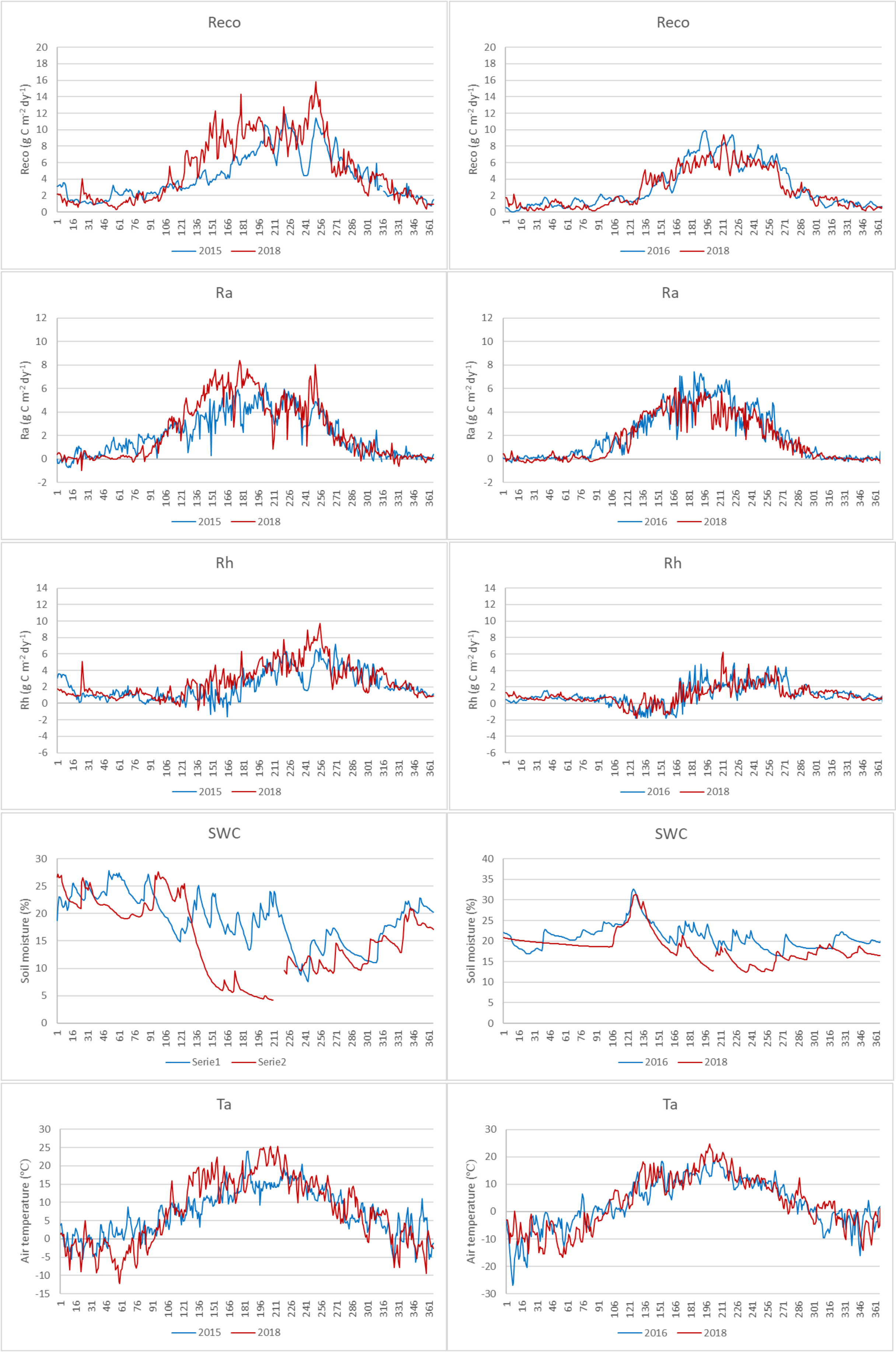

Figure S2e. Seasonal variation of respiration components, soil moisture and air temperature at Norunda and Svartberget.

Sorø

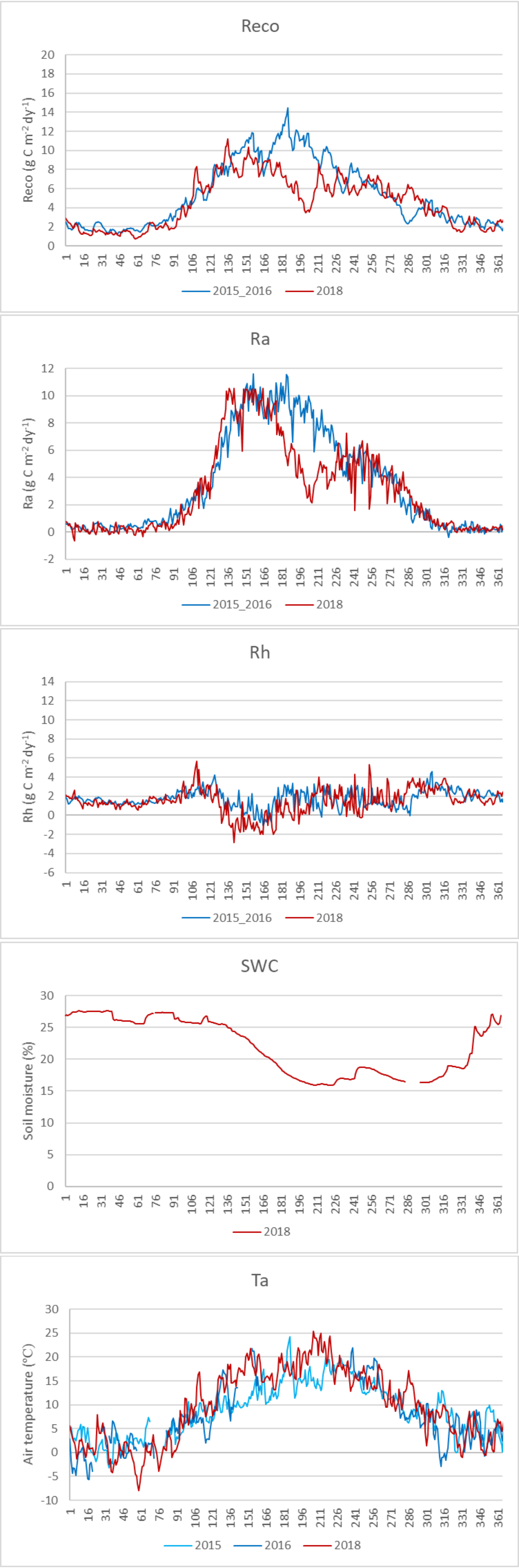

Figure S2f. Seasonal variation of respiration components, soil moisture and air temperature at Sorø. Soil moisture is lacking in 2015 and 2016.

Table S3. Site description.

Site name (acronym)	Lat., Long.	Species	Tree height (m)[1]	Stand age (yrs)	Leaf area index[2]	Above ground C-stock (g m^{-2})	Soil C-stock (0-100 cm) g m^{-2})	C/N ratio in OH-layer	Management method
Hyltemossa (Htm)	56°06′N, 13°25′E	Norway Spruce (Picea abies (L.) H.Karst.)	20.6	31 - 36	3.9 - 4.3	17600	9553.6 ± 3346.3	23.8 ± 1.74	Rotation
Hyytiälä (Hyy)	61°51′N, 24°18′E	Scots Pine (Pinus sylvestris L., 60 %), Norway Spruce (Picea abies (L.) H.Karst., 25%), Birch (Betula spec., 15%)	20	56	3	6000	6500	34	Rotation
Kenttärova (Ken)	67°59′N, 24°15′E	Norway Spruce (Picea abies (L.) H.Karst.)	14.5	80 - 240	2.0	3000	N/A	N/A	Not managed for the last 50 years
Norunda (Nor)	60°05′N, 17°29′E	Scots Pine (Pinus sylvestris L., 62 %), Norway Spruce (Picea abies (L.) H.Karst., 37%), Birch (Betula spec., 1%)	30	75 - 130	2.7	9200	22390	25.1	Rotation
Rosinedal (Ros)	64°10′N, 19°45′E	Scots Pine (Pinus sylvestris L.)	19.5	90	2,7	6000	N/A	N/A	Rotation
Rumperöd (Rum)	56°20′N, 14°07′E	Norway Spruce (Picea abies (L.) H.Karst., 56%), Scots Pine (Pinus sylvestris L., 16 %), European Beech (Fagus sylvatica L., 11%), Oak (Quercus robur L., 8%), Birch (Betula spec., 7%), Rowan and Alder (Sorbus and Alnus spec., 3%)	28	1 - 100	5 - 6	14 800 ± 7000	35900	25.9	Continuous
Skogaryd (Skg)	58°22′N, 12°09′E	Norway Spruce (Picea abies (L.) H.Karst.)	28	60	6-7	15000	55000	18 ± 1.2	Rotation
Soontaga (Son)	58°01′N, 26°14′E	Scots Pine (Pinus sylvestris L., 85 %), Norway Spruce (Picea abies (L.) H.Karst., 15%)	31	60 - 210	N/A	10173 ± 2250	10123 ± 691	35.1 - 35.8	Continuous
Sorø (Sor)	55°29′N, 11°38′E	European Beech (Fagus sylvatica L.)	30	98	5	9886 ± 2 32	9254± 2809	19 - 38	Continuous

Svartberget (Svb)	64°15′N, 19°46′E	Scots Pine (Pinus sylvestris L., 61 %), Norway Spruce (Picea abies (L.) H.Karst., 34%), Birch (Betula spec., 5%)	20	100	3,3	5600	N/A	49.5	Rotation
Värriö (Var)	67°45′N, 29°37′E	Scots Pine (Pinus sylvestris L.)	12	65 (area burnt c. 1950)	1.3	2000	2250	35	Natural

[1]The height of the upper 10% distribution of trees within footprint

[2]Projected leaf area per unit ground area

Table S4. Flux instrumentation

Name	Height of EC system (m)	Gas analyzer	Sonic anemometer	Reference publication/Web site
Hyltemossa	27	Li-7200, LI-COR Inc., USA	Gill HS-50, Gill Instruments Ltd., UK	http://www.icos-sweden.se/station_hyltemossa.html
Hyytiälä	27	Li-7200, LI-COR Inc., USA	Gill HS-50, Gill Instruments Ltd., UK	Kolari et al., 2009
Kenttärova	23	LI7000, LI-COR Inc., USA	USA-1, Metek GmbH, Germany	Aurela et al., 2015
Norunda	36	Li-7200, LI-COR Inc., USA	Gill HS-50, Gill Instruments Ltd., UK	http://www.icos-sweden.se/station_norunda.html
Rosinedal	21.5	Li-7200, LI-COR Inc., USA	Gill R3-100, Gill Instruments Ltd., UK	Jocher et al., 2017
Rumperöd	35.0	EC155, Campbell Scientific, USA	CSAT3A, Campbell Scientific, USA	N/A
Skogaryd	33	EC155, Campbell Scientific, USA	CSAT3A, Campbell Scientific, USA	https://gvc.gu.se/english/research/skogaryd/subsite-6
Soontaga	39	Li-7200, LI-COR Inc., USA	uSonic-3 Class A, Metek GmbH, Germany	N/A
Sorø	43	Li-7000, LI-COR Inc., USA	Gill HS-50, Gill Instruments Ltd., UK	Pilegaard et al., 2011
Svartberget	32.5	Li-7200, LI-COR Inc., USA	uSonic3 Class-A, Metek GmbH, Germany	Chi et al., 2019
Värriö	16.6	Li-7200, LI-COR Inc., USA	USA-1, Metek GmbH, Germany	Kulmala et al., 2019

References

Aurela M., Lohila A., Tuovinen J-P., Hatakka J., Penttilä T., Laurila T. (2015) Carbon dioxide and energy flux measurements in four northern-boreal ecosystems at Pallas. Boreal Environment Research, 20, 455-473.

Chi, J., Nilsson, M.B., Kljun, N., Wallerman, J., Fransson, J.E.S., Laudon, J., Lundmark, T., Peichl, M. (2019) The carbon balance of a managed boreal landscape measured from a tall tower in northern Sweden. Agricultural and Forest Meteorology, 274, 29-41. https://doi.org/10.1016/j.agrformet.2019.04.010

Jocher, G., Ottosson Löfvenius, M., De Simon, G., Hörnlund, T., Linder, S., Lundmark, T., Marshall, J., Näsholm, T., Tarvainen, L. et al. (2017) Apparent winter CO2 uptake by a boreal forest due to decoupling. Agricultural and Forest Meteorology, 232, 23-34. doi.org/10.1016/j.agrformet.2016.08.002.

Kolari, P., Kulmala, L., Pumpanen, J., Launiainen, S., Ilvesniemi, H., Hari, P., Nikinmaa, E. (2009) CO_2 exchange and component CO_2 fluxes of a boreal Scots pine forest. Boreal Env. Res. 14: 761–783.

Kulmala, L., Pumpanen, J., Kolari, P., Dengel, S., Berninger, F., Köster, K., Matkala, L., Vanhatalo, A., Vesala, T., Bäck, J. (2019) Inter- and intra-annual dynamics of photosynthesis differ between forest floor vegetation and tree canopy in a subarctic Scots pine stand. *Agricultural and Forest Meteorology, 271,* 1-11. doi.org/10.1016/j.agrformet.2019.02.029

Pilegaard, K., Ibrom, A., Courtney, M.S., Hummelshøj, P., Jensen, N.O. (2011) Increasing net CO2 uptake by a Danish beech forest during the period from 1996 to 2009. Agricultural and Forest Meteorology 151: 934–946.

Vicente-Serrano S.M., Santiago Beguería, Juan I. López-Moreno, (2010) A Multi-scalar drought index sensitive to global warming: The Standardized Precipitation Evapotranspiration Index - SPEI. Journal of Climate 23: 1696-1718.
